# Effect of sizes of vinyl modified narrow-dispersed silica cross-linker on the mechanical properties of acrylamide based hydrogel

**DOI:** 10.1038/s41598-023-32185-4

**Published:** 2023-03-29

**Authors:** Md. Rezaul Karim, Mohammad Harun-Ur-Rashid, Abu Bin Imran

**Affiliations:** 1 Department of Chemistry, Bangladesh University of Engineering and Technology, Dhaka, 1000 Bangladesh; 2 Department of Chemistry, Hajee Mohammad Danesh Science and Technology University, Dinajpur, 5200 Bangladesh; 3 Department of Chemistry, International University of Business Agriculture and Technology, Dhaka, 1230 Bangladesh

**Keywords:** Medical research, Chemistry, Materials science, Nanoscience and technology

## Abstract

Polymeric hydrogel with the incorporation of nano to submicro-meter sized materials forms an exhilarating new generation of composite hydrogels. Most of the applications of hydrogels are in aqueous environments in which they swell to a very high degree. This emanates from low density of the polymer chains, making them highly inferior in terms of physical strength and their prospective applications. In order to address the weak mechanical properties, hydrogels have successfully prepared with high tensile strength and toughness by reinforcing the acrylamide (AAm) network with 3-methacryloxypropyltrimethoxysilane (MPTS) modified silica particles (MSiO_2_) as chemical cross-linker. The MSiO_2_ cross-linkers are prepared from narrow-dispersed silica particles (SiO_2_) of 100 nm, 200 nm, and 300 nm diameters to investigate the effect of cross-linker sizes on the mechanical strengths of hydrogels. The presence of MSiO_2_ remarkably increases the stretching ability and toughness of hydrogels compared to conventional hydrogels. The tensile strength, toughness, and Young’s modulus of the hydrogel decrease from 30 to 11 kPa, 409 to 231 kJ/m^3^, and 0.16 to 0.11 kPa, respectively, while the SiO_2_ particle size increase from 100 to 300 nm and the concentration of AAm and MSiO_2_ (%) are kept constant. The compressive strength and toughness of the hydrogel decrease from 34 to 18 kPa and 6 to 4 kJ/m^3^, respectively, but the Young’s modulus increases from 0.11 to 0.19 kPa. This work is excellent proof of regulating mechanical strength of hydrogel by adjusting the particle size of MSiO_2_ cross-linkers.

## Introduction

Hydrogels are three-dimensional cross-linked polymeric networks containing water or biological fluids in large amounts in their network, thus becoming swollen^1–6^. Generally, such extremely hydrated polymer structures exhibit both elastic and viscous behavior when deformed and resemble the structure of biological tissue. They have been drawing considerable attention from scientists and technologists because of their versatile and unique properties for multifaceted applications. For pragmatic applications, appropriate mechanical strengths are required for hydrogels. Unfortunately, in most cases, conventional hydrogels possess inferior poor mechanical strength due to several reasons, such as mesh size and inhomogeneous distribution of cross-linking throughout the gel network^7–9^. Because of this, scientists are constantly expanding their expertise, investing more time, and developing new techniques to create hydrogels that are both stretchable and mechanically robust for use in a variety of multidimensional applications.

Chemical cross-linking, adopted to fabricate first-generation conventional hydrogels, was unduly weak and brittle. The most effective design principle was based on constructing a potential energy dissipation model in the gel matrix by maneuvering sacrificial or reversible bonds that avert crack extension and damage under strain. Second-generation hydrogels with high Young’s modulus and tensile strengths have been developed by modifying the gel network structure to induce energy dissipative mechanisms at the molecular level^10^. Several smart and effective techniques have already been employed to increase the mechanical strength of hydrogels keeping all other desirable properties unchanged. It is worth mentioning that topological hydrogels^11^, slide-ring hydrogels^12^, nanocomposite (NC) hydrogels^13^, double-network hydrogels^14^, and macromolecular microsphere hydrogels^15^ are the most appropriate examples of such mechanically strong hydrogels. Among these, the fabrication of NC hydrogels has fascinated multifarious research interests paid particular attention to improving the mechanical properties of hydrogels and amplifying the scope of their applications.

Nanoparticles such as nanospheres, nanosheets, and nanotubes have been successfully incorporated into hydrogels where the polymer and nanofillers interaction is usually established through several mechanisms such as hydrogen bonding, van der Waals forces, π–π stacking, electrostatic interactions, and covalent bonding. The NC hydrogels with improved toughness and mechanical strength are formulated by either previously mentioned forces or by their combinations. The distinctive polymeric chain length and high molecular weight of the polymer network make the NC hydrogels unique. Each of the nanoparticles is associated with several polymeric chains. As a result, if any chain gets detached from the filler nanoparticle, there would be a rest of the adjacent chains to hold up the increased load without cracking and provide the gels with high stretchability. Well-dispersed, rigid, and surface-functionalized inorganic nanoparticles have been widely applied to significantly improve the strength or toughness of polymeric structure^16^. Most of the reported NC hydrogels have been formulated through the grafting of polymers onto the surface of inorganic components utilizing weak ionic forces, which may easily disrupt at harsh conditions or even in a suitable solvent. Some of the published reports^17–21^ prepared hydrogels by utilizing SiO_2_ nanoparticles as cross-linker, but none of the groups focused on the influence of the size of SiO_2_ nanoparticles on the mechanical properties of hydrogels.

Jamali et al. formulated pH-sensitive hydrogel microspheres of AAm and Acrylic Acid (AAc) monomers by introducing SiO_2_ nanoparticles via inverse suspension polymerization^22^. Linn et al. used SiO_2_ nanoparticles to improve weak mechanical properties of poly(*N,N*-dimethylacrylamide) (PDMA) hydrogels through the self-cross-linking method but they did not mention the features of SiO_2_ nanoparticles^23^. Genovese et al. reported the effect of particle size (small: < 125 µm, and large: 125–850 µm) on mechanical properties and structure of methoxyl pectin/apple particles composite gels^24^. Rheological data of this report showed that increasing the concentration of small particles enhanced the elastic modulus of the composite gels. Yang and his group reported about physical hydrogels prepared by SiO_2_ nanoparticle surface in situ polymerization^25^. They prepared a series of poly acrylic acid (PAAc) hydrogels where covalently grafted chains constructed the hydrogel networks, and SiO_2_ nanoparticles acted as analogous cross-linking centers. The content and diameter of SiO_2_ nanoparticles significantly altered the physical properties of hydrogels. They applied SiO_2_ nanoparticles of wide diameter variations, such as 74 nm to 772 nm, and showed the relationship between the swelling ratio of hydrogels and the diameter of SiO_2_ nanoparticles. Chang et al. investigated the impact of the addition of nanoparticles on the mechanical properties of hydrogels^26^. The incorporation of nanoparticles made double-network hydrogel stronger with higher elastic moduli but failed to clearly demonstrate particle size’s influence. Levin et al. compared the improvement of the rigidity of PAAm and PDMA hydrogels by adding a tiny amount of SiO_2_ nanoparticles and established that high concentrations of SiO_2_ nanoparticles increased the toughness of both hydrogels but did not show any data related to the influence of particle size^27^. Azimi et al. prepared sulfonated polyacrylamide (SPAM)/chromium(III) acetate-based NC hydrogels and improved the mechanical properties by incorporating the various concentrations and sizes of SiO_2_ nanoparticles^28^. We recently reported a facile strategy for designing SiO_2_ based single polymer network hydrogel via strong covalent interactions between SiO_2_ and polymer in which the cross-linking density and inter-crosslinking distance can be regulated exclusively to enhance their mechanical properties^29^. In the previously published literature studies, it has been realized to establish the correlation between the mechanical strength of AAm based hydrogels and the diameters of inorganic cross-linkers.

**Figure 1:**
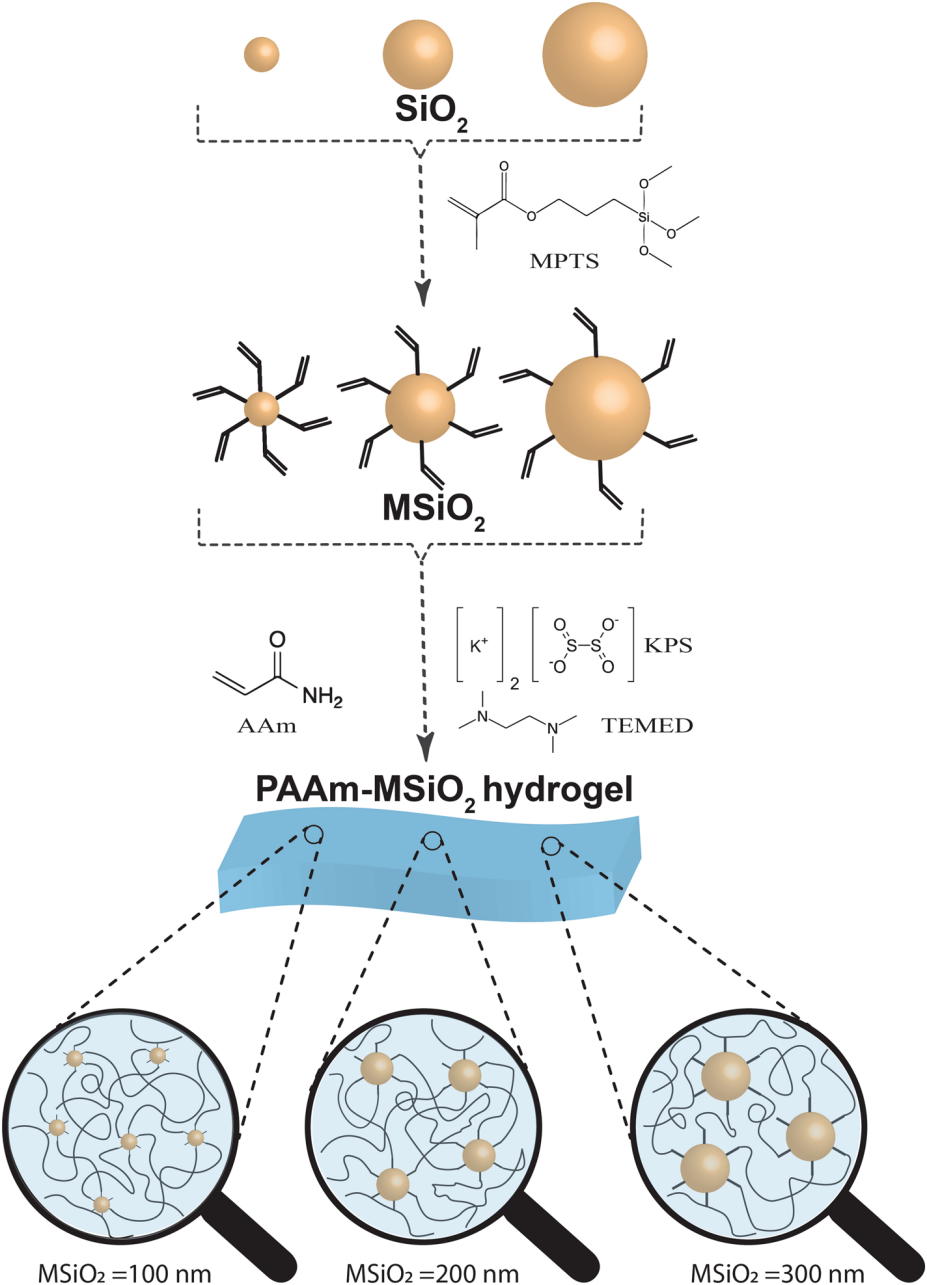
Schematic representation of the fabrication of PAAm-MSiO2 hydrogel prepared by varying particle sizes of MSiO_2_ (100 nm, 200 nm, 300 nm) cross-linker.

With the aim of achieving the above-mentioned goal, a straightforward approach have been presented here for preparing SiO_2_-based hydrogels through the implementation of strong covalent interactions between organic polymer and inorganic particles in which the degree and length of cross-linking can be synchronized exclusively to improve the mechanical properties of gel. A nano to sub micrometer sized SiO_2_ as inorganic particles has been chosen because of its biocompatibility, strong surface binding energy, superior chemical reactivity and stability, nontoxicity, large surface area, good absorbency, and versatile functionalization. SiO_2_ particles with diameters of 100 nm, 200 nm, and 300 nm will be modified by MPTS so that they could be used as chemical cross-linkers during free radical polymerization (Fig. 1). The effect of the size of the SiO_2_ particles on the mechanical properties of polyacrylamide-MSiO2 (PAAm-MSiO2) hydrogels will be studied.

## Experimental section

### Materials

AAm, *N,N′*-methylenebisacrylamide (BIS), potassium persulfate (KPS), and tetramethylethylenediamine (TEMED) were used as principal monomer, conventional cross-linker, initiator, and accelerator, respectively, for free radical polymerization. Additionally, MPTS as SiO_2_ particle modifier, acetic acid as pH adjuster, ethanol, and deionized water as solvents were used in this work. All the analytical grade chemicals were purchased from Sigma-Aldrich and used without further purification. The narrow-dispersed SiO_2_ particles having different diameters (100 nm, 200 nm, and 300 nm) were purchased from Nippon Shokubai, Japan, and used as received.

### Modification of SiO_2_ particles

At first, 10 (wt%) MPTS was hydrolyzed in ethanol–water (9:1) solution, and the pH was maintained at 3–4 by using an acetic acid solution (0.1 mol L^−1^). The mixture of hydrophilic SiO_2_ was then added to the solution and heated at 60–70 °C in an oil bath under magnetic stirring at 500 rpm for half an hour. Subsequently, the silanized SiO_2_ were separated by centrifugation (Centrifuge machine, Hettich, Universal 16A) and submersed in deionized water for 24 h. To eliminate the influence of physical adsorption, the total product was washed with deionized water several times and dried at 60 °C for 24 h until reaching a constant weight to obtain MSiO_2_.

### Synthesis of PAAm-BIS and PAAm-MSiO_2_ hydrogels

PAAm-MSiO_2_ hydrogels were synthesized by free radical polymerization at room temperature using KPS, TEMED, and MSiO_2_ particles as initiator, accelerator, and cross-linker, respectively. Initially, variable weight percentages of MSiO_2_ with respect to AAm were dispersed properly in deionized water. Then a predetermined molar concentration of AAm was added in the dispersion under an inert nitrogen atmosphere. The mixture was poured into two different types of mold, made up of two glass slides kept apart by 1–4 mm Teflon spacers and test tubes with varying diameters. The flat glass mold and test tube were kept in an ice bath while mixing the precursors. The polymerization was done at room temperature for 24 h. The PAAm-MSiO_2_ hydrogels were prepared by changing the concentration of AAm (3 M, 4 M, and 5 M) and keeping fixed the amount of MSiO_2_ with different particle diameters (100 nm, 200 nm, and 300 nm). The conventional PAAm-BIS hydrogels were fabricated using different concentrations of traditional cross-linker BIS. The formulations to fabricate various types of PAAm-MSiO_2_, PAAm-BIS, and PAAm-BIS-MSiO_2_ hydrogels by varying the concentration of monomers and other gel precursors such as cross-linker, initiator, and solvent have been listed in Table 1.

**Table 1:** The formulations of different types of PAAm-BIS and PAAm-MSiO_2_ hydrogels.

Hydrogel code	AAm (M)	BIS (%)	MSiO_2_ (%)	MSiO_2_ size (nm)
PAAm-BIS 2/1.0	2.00	1.00%	0	**–**
PAAm-BIS 4/1.0	4.00	1.00%	0	**–**
PAAm-MSiO_2_ 3/0.75 (100 nm)	3.00	0	0.75	100
PAAm-MSiO_2_ 4/0.75 (100 nm)	4.00	0	0.75	100
PAAm-MSiO_2_ 5/0.75 (100 nm)	5.00	0	0.75	100
PAAm-MSiO_2_ 3/0.75 (200 nm)	3.00	0	0.75	200
PAAm-MSiO_2_ 4/0.50 (200 nm)	4.00	0	0.50	200
PAAm-MSiO_2_ 4/0.75 (200 nm)	4.00	0	0.75	200
PAAm-MSiO_2_ 4/1.00 (200 nm)	4.00	0	1.00	200
PAAm-MSiO_2_ 4/1.25 (200 nm)	4.00	0	1.25	200
PAAm-MSiO_2_ 5/0.75 (200 nm)	5.00	0	0.75	200
PAAm-MSiO_2_ 3/0.75 (300 nm)	3.00	0	0.75	300
PAAm-MSiO_2_ 4/0.75 (300 nm)	4.00	0	0.75	300
PAAm-MSiO_2_ 5/0.75 (300 nm)	5.00	0	0.75	300

### Fourier transform infrared (FT-IR) spectroscopic analysis

The functional groups analysis on the surface of SiO_2_ particles was investigated by FT-IR spectrophotometer (FT-IR-8400, Shimadzu, Japan). The SiO_2_ and MSiO_2_ were analyzed by FT-IR spectrophotometer in the region of 4000 cm^–1^ to 470 cm^–1^. SiO_2_ and MSiO_2_ were dried in an oven at 60 °C, and a small portion of the samples was taken into vials. The solid samples were ground in mortar by pestle and mixed with moisture-free pure KBr (Sigma-Aldrich, Germany) crystals, and the powder mixture was converted into pellets by pressing manually under a pressure of 8–10 tons. Finally, the pellet was positioned inside the sample chamber for FT-IR spectra measurements.

### Nuclear magnetic resonance (NMR) spectroscopic analysis

The modification of SiO_2_ was verified by observing the ^1^H-NMR spectra recorded by Bruker BPX-400 spectrometer (400 MHz). Deuterated chloroform (CDCl_3_) was used as a solvent, and tetramethylsilane (TMS) was used as an internal standard. The coupling constant (j) was in Hz, and all the chemical shifts (δ) relative to TMS peak and solvent peaks (7.28 ppm) were recorded in ppm. The abbreviations for singlet, doublet, triplet, and quartet are used as s, d, t, and q, respectively.

### Field emission scanning electron microscopy (FE-SEM)

The surface morphology of SiO_2_ and MSiO_2_ of different diameters was explored using FE-SEM (JSM-7600F, Tokyo, Japan). The selected samples of different characteristics were dried and coated with platinum by sputtering to ensure the conductivity of samples’ surfaces. The observation of sample surface by FE-SEM was carried out at an accelerating voltage of 5.0 kV.

### Energy dispersive X-ray spectroscopy (EDXS)

Elementary composition investigation of SiO_2_ and MSiO_2_ of different diameters was performed by EDXS connected to a microscope (FE-SEM; JSM-7600F, Tokyo, Japan). The sample preparation for EDXS analysis is similar to that of FE-SEM analysis procedure.

### Fiber optic ultraviolet–visible (UV–Vis) spectrophotometry

The transparency of prepared hydrogels was examined by measuring the transmittance spectra using fiber optic UV–Vis spectrometer (FLAME-T-XR1, Ocean Optics, Germany). The DH-2000-BAL was used as a balanced deuterium halogen light source. The transmittance spectra were measured in the 200–800 nm at 25 °C. The 2.0 mm thick hydrogel samples were positioned on the glass surface between the UV–Vis light source and detector perpendicular to each other.

### Mechanical properties for elongation and compression

The mechanical properties of hydrogels were assessed by a universal testing machine (UTM, TestResources, Model 100-P-250-12). Flat hydrogel samples with 2.0 mm in width and 10.0 mm in length were used for uniaxial tensile measurement.

The Young’s modulus was calculated from the slope of 10% strain in the stress–strain curve. The fracture toughness was calculated by integrating the area of the stress–strain curve of each sample. Cylindrical shaped hydrogels samples with 10–15 mm diameter and 4–8 mm thickness was selected for compressive stress–strain measurements. All the mechanical properties were measured at ambient temperature. The crosshead speed was fixed at 50 mm/min. Each sample was tested at least three times to check the reproducibility of the results.

## Results and discussion

### Confirmation of SiO_2_ particle modification

**Figure 2:**
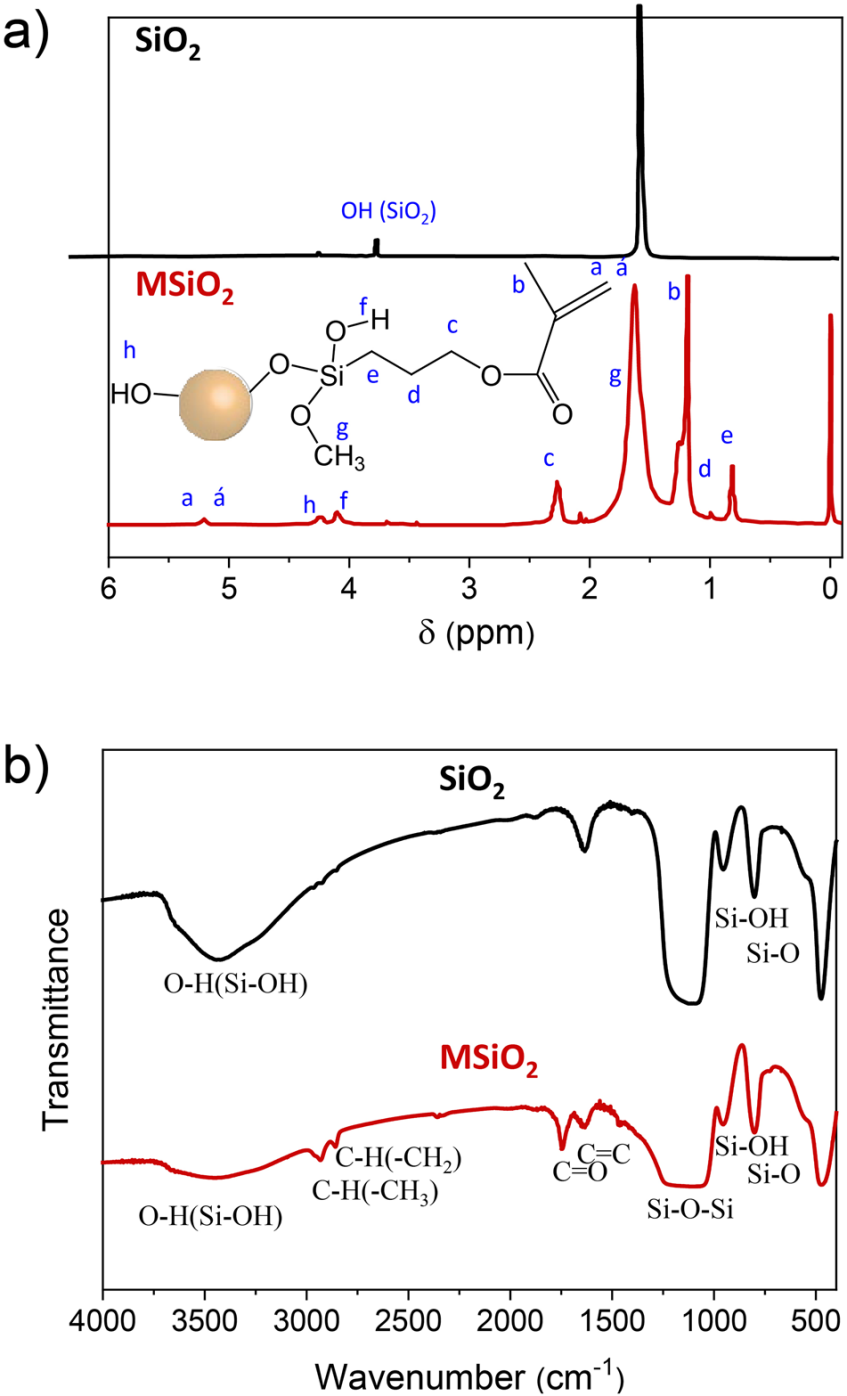
(**a**) ^1^H-NMR spectra of bare SiO_2_ 200 nm and MSiO_2_ 200 nm and (**b**) FT-IR spectra of bare SiO_2_ 200 nm and MSiO_2_ 200 nm.

The FTIR spectra confirmed that MPTS successfully modified with the bare SiO_2_. The peak at 1100 cm^−1^ for the stretching vibration of Si–O–Si, the peak at 950 cm^−1^ for Si–OH, the peak at 802 cm^−1^ for the stretching vibration of Si–O, and the peak at 470 cm^−1^ belonging to the vibration of Si–O groups were observed in the spectrum (Fig. 2). In MSiO_2_, a carboxyl group (C=O) peak at 1740 cm^−1^, and also peaks at 2957, 2882, and 1645 cm^−1^ which were because of C–H(–CH_3_), C–H(–CH_2_), and C=C stretching vibration, respectively, were observed. The appearance of all these peaks and bands indicated that the MPTS had been successfully grafted onto the surface of SiO_2_ particles. The decrease in peak intensity of the –OH band found in the spectrum of MSiO_2_ also indicates the successful incorporation of MPTS to –OH groups of SiO_2_. The presence of relevant characteristic signals of MPTS at δ = 5.28 ppm, 5.32 ppm (a, a′), 1.27 ppm (b), 2.35 ppm (c), 1.06 ppm (d) 0.9 ppm (e) in ^1^H-NMR spectrum (Fig. 2) produced by MSiO_2_ whereas all peaks were absent in bare SiO_2_. The above observations prove the successful modification of the SiO_2_ particles by MPTS and could be used as cross-linkers for gel preparation.

### Morphology investigation of SiO_2_ and MSiO_2_ particles

**Figure 3:**
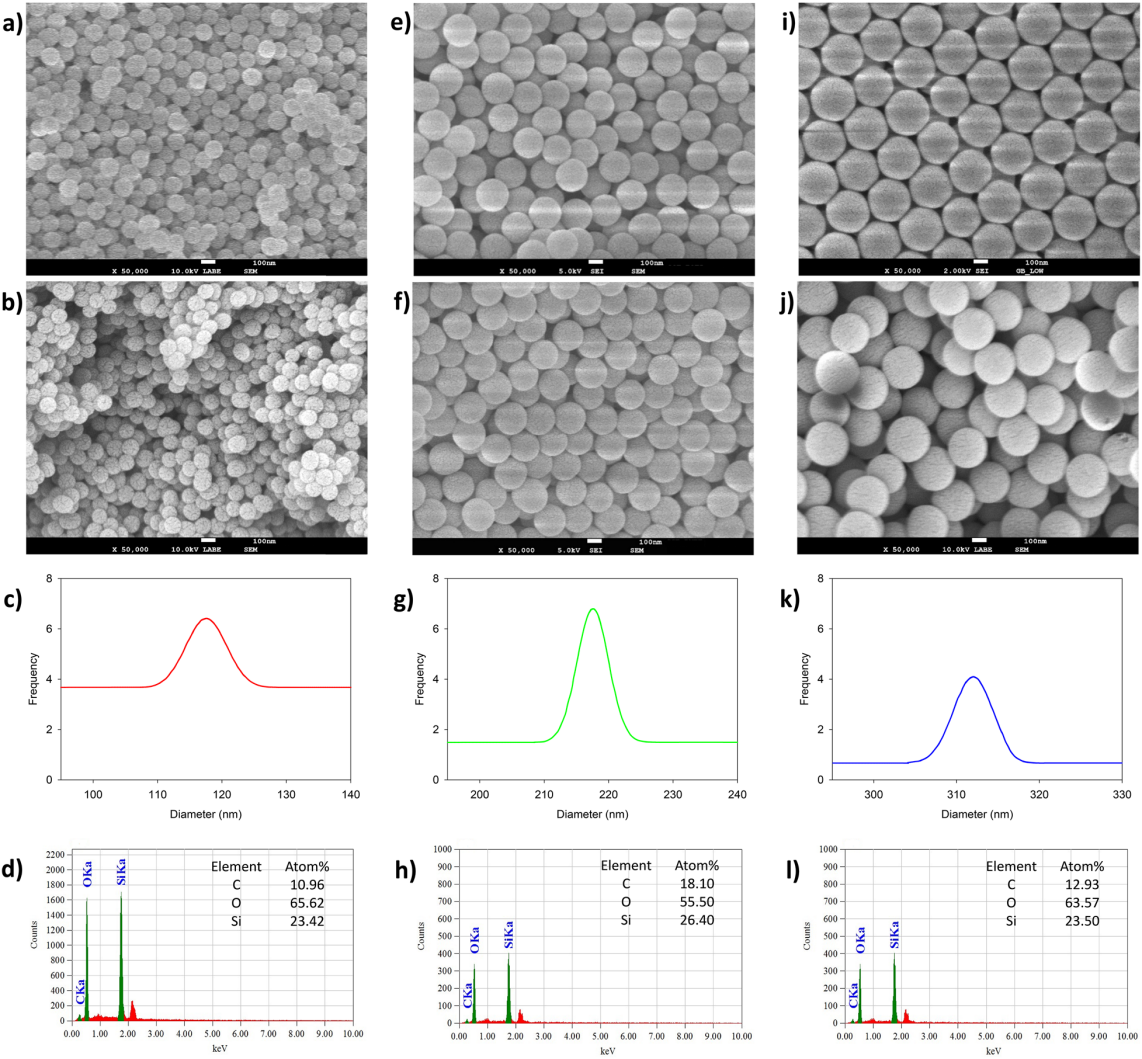
FE-SEM images of SiO_2_, MSiO_2_, particle size distribution curve of MSiO_2_, and EDXS spectra of MSiO_2_ having 100 nm (**a–d**), 200 nm (**e–h**), and 300 nm (**i–l**) diameters, respectively.

The surface morphologies of all bare SiO_2_ and MSiO_2_ (100 nm, 200 nm, and 300 nm) were analyzed by FE-SEM images. The spherical shape and narrow dispersity of all SiO_2_ and MSiO_2_ were confirmed from FE-SEM micrographs (Fig. 3). Finally, the EDXS spectra of MSiO_2_ particles validated the successful modification of bare SiO_2_ particles (Fig. 3). The size of SiO_2_ (100 nm) ranged from 100 to 115 nm and the average particle size was 110.5 ± 4.5 nm. The size of MSiO_2_ (100 nm) was ranging from 100 to 120 nm and the average particle size was 117.5 ± 5.0 nm. The particle sizes were slightly increased from bare SiO_2_ to MSiO_2_ after a successful vinyl groups incorporation, but they did not induce any significant changes in the particle size distribution. Similar surface morphologies and average particle size variations were observed in cases of SiO_2_ (200 nm/300 nm) and MSiO_2_ (200 nm/300 nm). The bare SiO_2_ (200 nm) sizes ranged from 204 to 213 nm, and average particle size was 208.5 ± 3.0 nm. The size of MSiO_2_ (200 nm) ranged from 205 to 230 nm, and average particle size was 216.5 ± 7.2 nm. The bare SiO_2_ (300 nm) ranged from 295 to 315 nm, and the average particle size was 302.5 ± 5.3 nm. The size of MSiO_2_ (300 nm) ranged from 300 to 318 nm, and the average particle size was 311.5 ± 5.5 nm. The EDXS data provided information about the specific chemical elements present in bare SiO_2_ to MSiO_2_ particles. The existence of carbon, silicon, and oxygen in MSiO_2_ was confirmed by EDXS spectra generated by MPTS modifier.

The average zeta potential value for bare SiO_2_ was in the range of − 47.9 to − 53.1 mV and that of MSiO_2_ was − 47.1 to − 57.1 mV, which suggested that those particles were highly stable in water. Generally, colloids with a zeta potential value of more than ± 30 mV are considered stable.

### Transparency of PAAm-MSiO_2_ hydrogels

**Figure 4:**
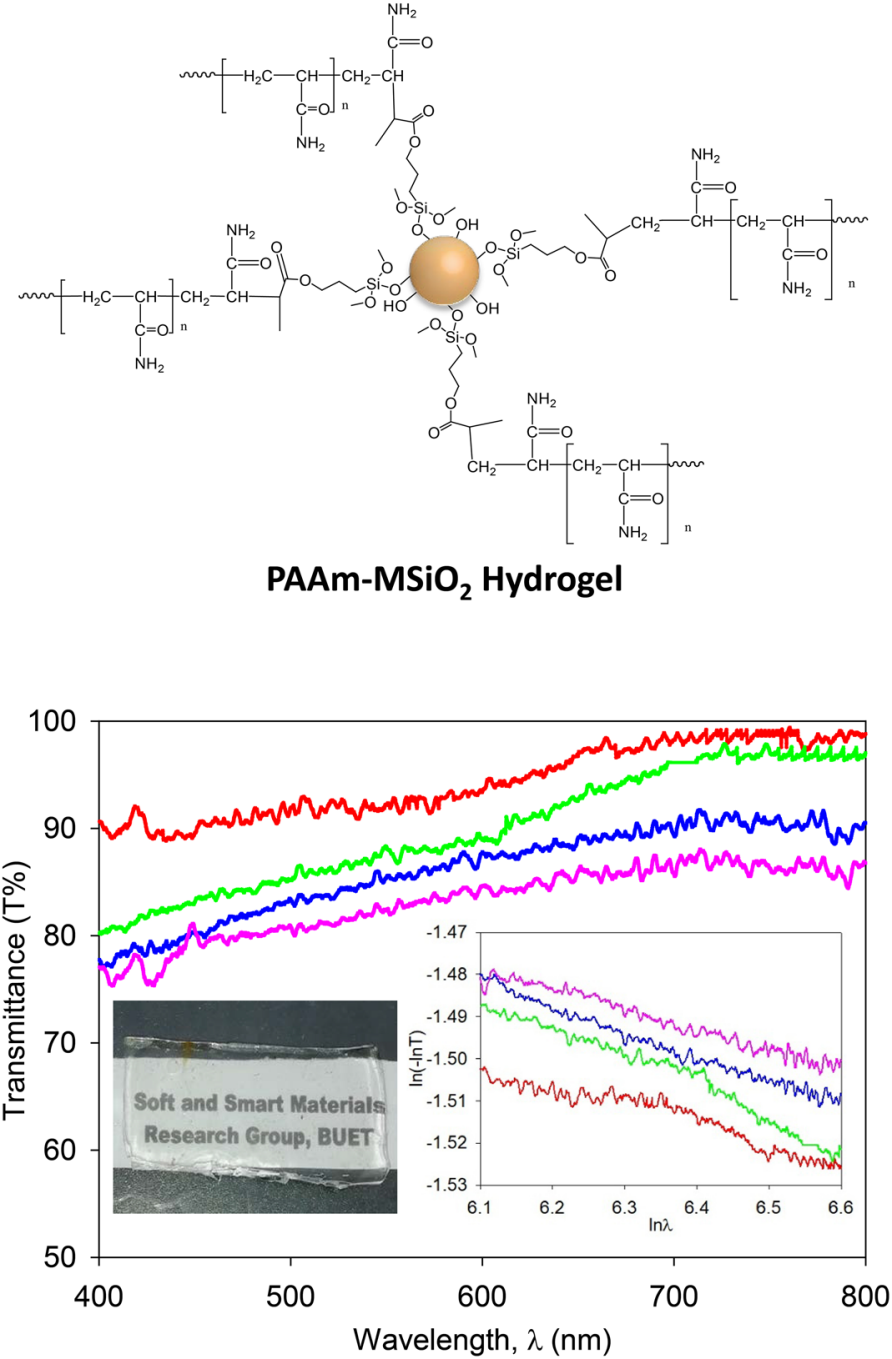
Proposed structure of PAAm-MSiO_2_ hydrogel (top), the transmittance spectra of highly transparent hydrogel (bottom), transparent gel sample (inset-left), and the plots of ln(-lnT) versus lnλ (inset-right). In the graph, red line, green line, blue line, and purple line represent PAAm (4 M)-MSiO_2_ 0.50%, PAAm (4 M)-MSiO_2_ 0.75%, PAAm (4 M)-MSiO_2_ 1.00%, and PAAm (4 M)-MSiO_2_ 1.25% hydrogels, respectively.

The PAAm-MSiO_2_ hydrogels were highly transparent throughout the range of visible light (400 nm to 800 nm), and the transparency of the hydrogels was inversely proportional to the amount of MSiO_2_ cross-linker (Fig. 4). The PAAm-MSiO_2_ hydrogels made of 0.50% cross-linker showed more than 90% transmittance, while the transparency of PAAm-MSiO_2_ made of 1.25% cross-linker was around 75%. The high transparency of PAAm-MSiO_2_ hydrogel confirmed the homogenous dispersion of cross-linkers. The homogeneous size, shape, and stable dispersion in water of MSiO_2_ cross-linkers increased the possibilities of uniform polymer networks formation. The Rayleigh scattering occurs when light passes through a medium where the size of the agglomerations is smaller than the wavelength of visible light. The slopes for PAAm (4 M)-MSiO_2_ 0.50%, PAAm (4 M)-MSiO_2_ 0.75%, PAAm (4 M)-MSiO_2_ 1.00%, and PAAm (4 M)-MSiO_2_ 1.25% hydrogels (inset of Fig. 4 bottom-right) from ln(**− **lnT) versus ln λ plots are − 0.070, − 0.064, − 0.053, and − 0.044, respectively. The slopes of relevant trend lines imply that the hydrogels were slightly deviating from the Rayleigh scattering of light, which is inversely proportional to the fourth power of the wavelength^30^. Mie scattering of light was expected in the case of our hydrogels as the sizes of the aggregated clusters were similar to or slightly bigger than the wavelengths of visible light. Though all the PAAm-MSiO_2_ hydrogels produced Mie scattering of light, the size of clusters present in gels composed of a lower amount MSiO_2_ cross-linker was larger than that of gels composed of a higher amount of MSiO_2_ cross-linker.

The reinforcing effect of SiO_2_ particles can be adjusted finely and precisely by several other parameters, such as the nature of the particles, matrix properties, aspect ratio of particles, particle volume fraction, particle size, particle orientation and distribution, particle structural variations, and strength of interactions across the particles and polymer chains^31^. The reinforcement mechanism in polymer micro composites is similar to that present in nanocomposites except for the effect of particle size and structural parameters, which are expected to play a vital role in the mechanical behavior of nanocomposites. Micromechanical propositions such as the Halpin–Tsai^32^ and Mori–Tanaka^33^ approaches, extensively applied for micro composites, have been proposed by many researchers to evaluate the overall elastic stiffness of nanocomposite polymeric materials^34^. Researchers conjectured that the size of filler particles had an effect on the properties of composite materials, but reliable experimental data are still not available to support the concluding remark. Few research groups examined numerically nanocomposite properties applying spherical fillers (SiO_2_ particles) and observed that the composites properties could be intensified by decreasing the particle size^35,36^. Keeping in mind the scarcity of research data that reflects particle size’s influence on the reinforcement of PAAm-MSiO_2_ hydrogels, PAAm-MSiO_2_ hydrogels have been formulated by varying the diameters of MSiO_2_ cross-linker and investigated the particle size effect on mechanical properties of gels. The effect of concentration of both monomer and MSiO_2_ cross-linker on their mechanical strengths is also examined in detail.

### Superior mechanical properties of PAAm-MSiO_2_ hydrogels than PAAm-BIS hydrogels

**Figure 5:**
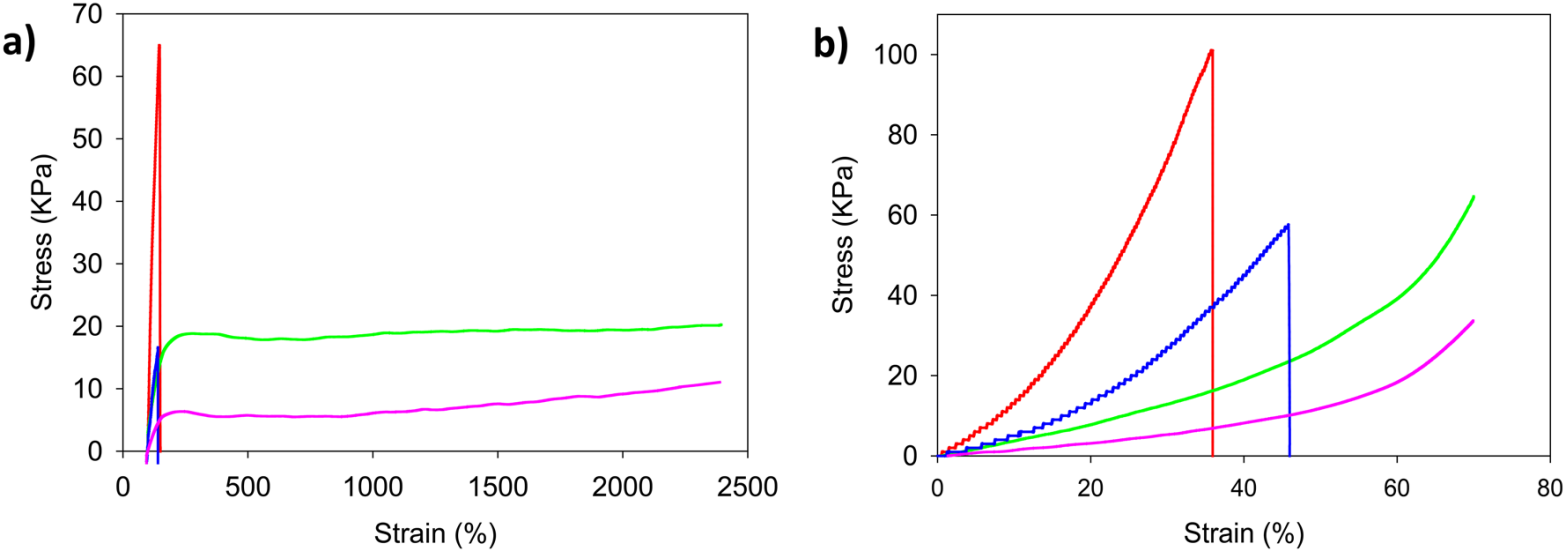
(**a**) Stress–strain curves of PAAm-MSiO_2_ and PAAm-BIS hydrogels prepared by varying the BIS and MSiO_2_ under uniaxial tension and (**b**) stress–strain curves of PAAm-MSiO_2_ and PAAm-BIS hydrogels prepared by varying the concentration of BIS and MSiO_2_ under uniaxial compression. In the graph, red line, green line, blue line, and purple line represent PAAm (4 M)-BIS 1.00%, PAAm (4 M)-MSiO_2_ 1.00%, PAAm (2 M)-BIS 1.00%, and PAAm (3 M)-MSiO_2_ 0.75% hydrogels, respectively.

PAAm-MSiO_2_ and PAAm-BIS hydrogels were notably different with respect to their mechanical properties during elongation (Fig. 5). The tensile strength and Young’s modulus of PAAm-MSiO_2_ were low compared with the mechanically toughened PAAm-BIS hydrogels. But PAAm-BIS hydrogels readily broke at lower applied stress during elongation, and its elongation at break was found in the 135% to 160% range. Due to the inhomogeneous cross-linking density in PAAm-BIS hydrogel, the short polymer chain length failed to dissipate energy. On the contrary, PAAm-MSiO_2_ hydrogel demonstrated an extended elongation range reaching up to 2400% of strain (maximum instrumental limit) without breaking (Table 2). The chemical bonding, physical interactions, and the rigorous dispersion of MSiO_2_ cross-linker across the polymer chains of hydrogel network provided much emancipation to dissipate energy under applied stress. The tensile strength, Young’s modulus, and toughness of PAAm-MSiO_2_ hydrogels changed with increasing MSiO_2_ content. At constant MSiO_2_ content, the tensile strength, Young’s modulus, and toughness increased with the AAm concentration (Fig. 5a). The tensile properties of PAAm-BIS hydrogels were also dependent on the AAm concentration. PAAm-MSiO_2_ hydrogels and PAAm-BIS hydrogels were remarkably different with regard to their compressive strength. Typical strain–stress curves for different weight percentages of cross-linker based hydrogels have been shown in Fig. 5b. It was observed that PAAm-BIS hydrogels readily broke at lower deformation by applying force and its break was in the range of 38–45%. Contrariwise, PAAm-MSiO_2_ hydrogel did not get broken under 70 percent deformation. The brittleness of typical PAAm-BIS hydrogels could be trounced by employing MSiO_2_ as cross-linker for fabricating PAAm-MSiO_2_ hydrogel with soft rubber-like functions. The higher concentration of MSiO_2_ cross-linker increased the strength of PAAm-MSiO_2_ hydrogel by creating well-built and strong interactions across the polymer chains and MSiO_2_ particles, resulting in an increase in Young’s modulus, toughness, and compressive strength.

**Table 2:** Comparison of tensile and compressive properties of PAAm-BIS and PAAm-MSiO_2_ hydrogels prepared by changing the concentration of AAm, BIS, and MSiO_2_.

Hydrogel code	Toughness (kJ/m^3^)		Tensile strength (kPa)	Compressive strength (kPa)	Young modulus (kPa)		Elongation at break (%)	
	Tension	Compression			Tension	Compression	Tension	Compression
PAAm (4 M)-BIS 1.00%	203	14	65.00	101	2.12	1.63	150	38
PAAm (4 M)-MSiO_2_ 1.00%	453*	14	20.99*	65	0.24	0.46	No break	No break
PAAm (2 M)-BIS 1.00%	41	10	18.00	57	0.44	0.66	144	45
PAAm (3 M)-MSiO_2_ 0.75%	231*	9	11.20*	26	0.11	0.11	No break	No break

*Due to the maximum limit of the instruments, the parameter has to be calculated after 24 times of elongation without a break in samples.

### Tensile and compressive strength of different PAAm-MSiO_2_ (100 nm, 200nnm, and 300 nm) hydrogels prepared by varying concentrations of AAm

**Figure 6:**
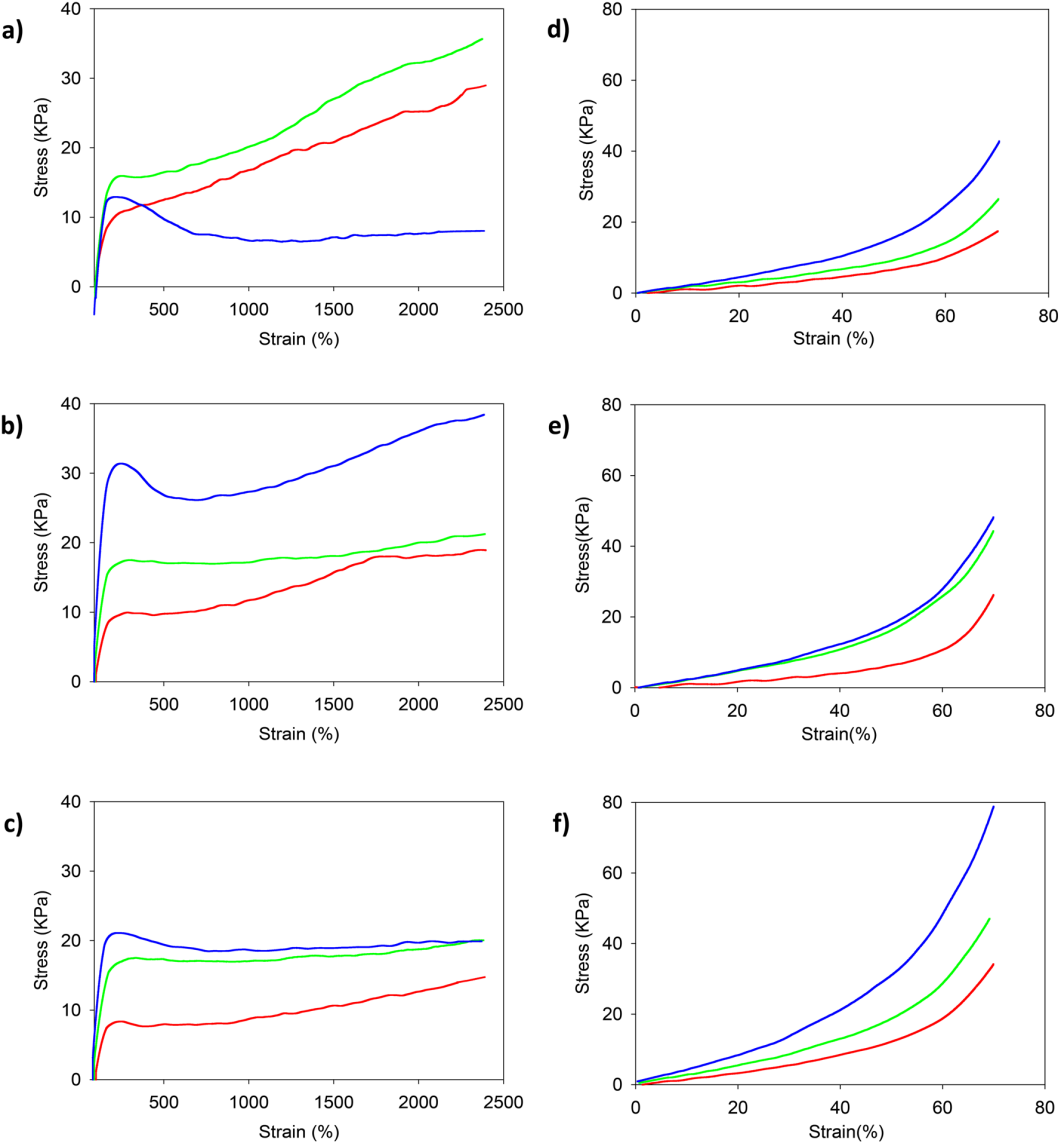
Tensile strength test results of PAAm-MSiO_2_ hydrogels prepared by 117.5 nm (**a**), 216.5 nm (**b**), and 311.5 nm (**c**) MSiO_2_ cross-linker and compressive strength test results of PAAm-MSiO_2_ hydrogels prepared by 117.5 nm (**d**), 216.5 nm (**e**), and 311.5 nm (**f**) MSiO_2_ cross-linker. In the graph, red line, green line, and blue line represent PAAm (3 M)-MSiO_2_ 0.75%, PAAm (4 M)-MSiO_2_ 0.75%, and PAAm (5 M)-MSiO_2_ 0.75% hydrogels, respectively.

Figure 6 shows the comparison of tensile and compressive strength of PAAm-MSiO_2_ NC hydrogels prepared by changing the concentration of AAm (3 M, 4 M, and 5 M) and the diameter of MSiO_2_ cross-linkers (117.5 nm, 216.5 nm, and 311.5 nm). The mechanical properties (toughness, tensile strength, and Young’s modulus) of PAAm-MSiO_2_ hydrogel significantly increased with the increase of AAm concentration under the constant weight percentage of MSiO_2_ cross-linker (Table 3). When the concentration of AAm increased, the entanglement of the polymer chains and the interaction between the AAm chain and MSiO_2_ cross-linker were also increased. The PAAm-MSiO_2_ hydrogel for the 3 M AAm concentration, the interaction between MSiO_2_ cross-linker and AAm chain was weaker than that of 4 M AAm hydrogel. As a result, it exhibited lower mechanical properties than that of 4 M hydrogels. But for the 5 M AAm concentration, it started to decrease the stress after reaching a certain point of stress. But for the other two hydrogels, the stress gradually increased after the yield point in the plastic region. Regardless of the direction in which the hydrogel was elongated, the hydrogel showed extraordinary extensibility that could reach 24 times the original length without a break. The stress–strain measurements could not be continued further due to the technical limitation of the instrument (Fig. 6a–c). The AAm concentration also significantly affected the compressive strength of PAAm-MSiO_2_ hydrogel. The toughness, compressive strength, and Young’s modulus of hydrogel significantly increased with increasing AAm concentration under the constant weight percentage of MSiO_2_ cross-linker. PAAm-MSiO_2_ hydrogel with high AAm concentration possessed higher compressive strength due to strong bonding between the polymer chain and MSiO_2_ particles and high entanglement of AAm polymer chains (Fig. 6d–f). Hence, when the AAm concentration was increased, the compressive strength, toughness, and Young’s modulus of hydrogel were increased (Table 4).

**Table 3:** Summary of tensile properties of PAAm-MSiO_2_ hydrogels prepared by varying concentrations of AAm (3 M, 4 M, and 5 M) and keeping the constant concentration (0.75 wt%) of MSiO_2_ particles having different diameters (117.5 nm, 216.5 nm, and 311.5 nm).

Hydrogel code	Young’s modulus (kPa)			Tensile strength (kPa)*			Toughness (kJ/m^3^)*		
	117.5 nm MSiO_2_	216.5 nm MSiO_2_	311.5 nm MSiO_2_	117.5 nm MSiO_2_	216.5 nm MSiO_2_	311.5 nm MSiO_2_	117.5 nm MSiO_2_	216.5 nm MSiO_2_	311.5 nm MSiO_2_
PAAm (3 M)-MSiO_2_ 0.75%	0.16	0.15	0.11	30	19	11	409	316	231
PAAm (4 M)-MSiO_2_ 0.75%	0.20	0.22	0.17	42	21	20	594	415	403
PAAm (5 M)-MSiO_2_ 0.75%	0.29	0.44	0.25	17	38	21	296	704	440

*Due to the maximum limit of the instruments, the parameter has to be calculated after 24 times of elongation without a break in samples.

**Table 4:** Summary of compressive properties of PAAm-MSiO_2_ hydrogels prepared by varying the concentration of AAm (3 M, 4 M, and 5 M) and keeping the constant concentration (0.75 wt%) of MSiO_2_ particles having different diameters (117.5 nm, 216.5 nm, and 311.5 nm).

Hydrogel code	Young’s modulus (kPa)			Compressive strength (kPa)			Toughness (kJ/m^3^)		
	117.5 nm MSiO_2_	216.5 nm MSiO_2_	311.5 nm MSiO_2_	117.5 nm MSiO_2_	216.5 nm MSiO_2_	311.5 nm MSiO_2_	117.5 nm MSiO_2_	216.5 nm MSiO_2_	311.5 nm MSiO_2_
PAAm (3 M)-MSiO_2_ 0.75% hydrogel	0.11	0.10	0.19	18	26	34	3.5	4.0	6.0
PAAm (4 M)-MSiO_2_ 0.75% hydrogel	0.15	0.18	0.27	26	44	46	5.2	9.0	9.0
PAAm (5 M)-MSiO_2_ 0.75% hydrogel	0.25	0.27	0.41	43	49	78	8.9	10.0	16.0

### Tensile strength of PAAm-MSiO_2_ hydrogels prepared by varying particle sizes of MSiO_2_ (117.5 nm, 216.5 nm, 311.5 nm)

**Figure 7:**
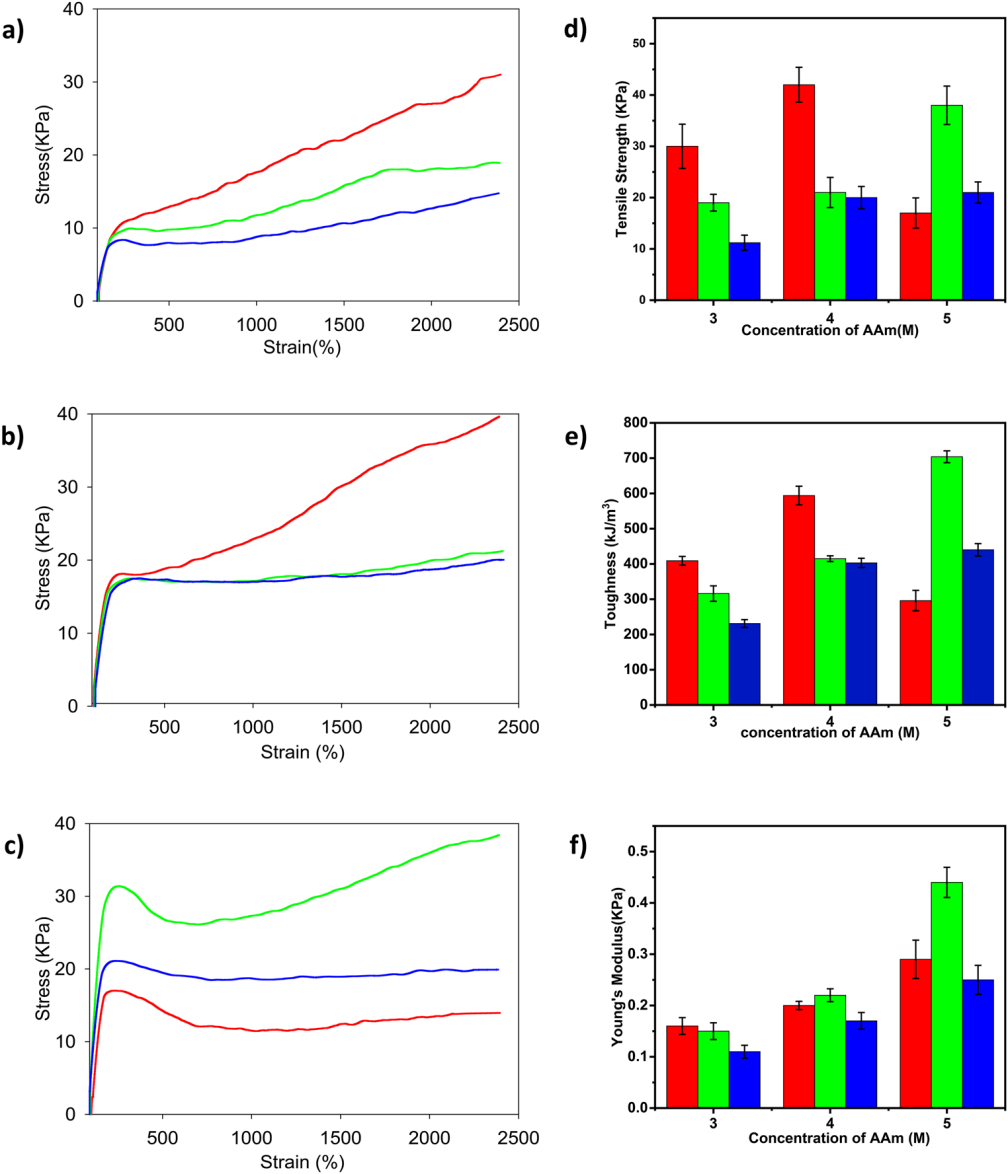
Stress–strain curves produced by tensile strength test of PAAm (3 M)-MSiO_2_ 0.75% (**a**), PAAm (4 M)-MSiO_2_ 0.75% (**b**), and PAAm (5 M)-MSiO_2_ 0.75% (**c**), hydrogels and comparison of tensile strengths (**d**), toughness (**e**), and Young’s modulus (**f**) of PAAm (3 M)-MSiO_2_ 0.75%, PAAm (4 M)-MSiO_2_ 0.75%, and PAAm (5 M)-MSiO_2_ 0.75 hydrogels. Red, green, and blue colors represent MSiO_2_ cross-linker having a diameter of 117.5 nm, 216.5 nm, and 311.5 nm, respectively.

The tensile properties of the hydrogel were increased with decreasing particle size of MSiO_2_ cross-linkers. The smaller SiO_2_ particles with a large surface area require more MPTS than the larger SiO_2_ particle during the modification process. As a result, MSiO_2_ cross-linkers with shorter diameters could form more cross-linking points with the polymer chains to form high cross-linking density compared to MSiO_2_ cross-linkers with longer diameters. Furthermore, the interaction between polymer chains and cross-linkers increased because of a high degree of cross-linking. Consequently, the strong interactions restricted the movement of the polymer chain during elongation and demonstrated higher tensile strength, Young’s modulus, and toughness. After maintaining a constant concentration of AAm and MSiO_2_ but by changing the size of SiO_2_ particle from 117.5 to 311.5 nm; the tensile strength, toughness, and elastic modulus of the hydrogel decreased from 30 to 11 kPa, 409 to 231 kJ/m^3^ and 0.16 to 0.11 kPa, respectively. This trend continued for different concentrations of AAm (Fig. 7, Table 5). When the concentration of AAm was increased from 3 to 4 M, the tensile strength, toughness, and Young’s modulus increased from 11.2 to 20 kPa, 231 to 403 kJ/m^3^, and 0.11 to 0.17 kPa, respectively. A similar tendency was found for the other diameters of MSiO_2_ cross-linker (Table 5). The mechanical properties for PAAm-MSiO_2_ hydrogel containing 5 M AAm follow slightly different trend.

**Table 5:** Comparison of tensile strength of PAAm-MSiO_2_ hydrogels prepared by varying the particle sizes of MSiO_2_ (117.5 nm, 216.5 nm, and 311.5 nm).

Hydrogel code	Toughness (kJ/m^3^)*	Tensile strength (kPa)*	Young’s modulus (kPa)	Elongation at break (%)
PAAm (3 M)-MSiO_2_ 117.5 nm	409	30.0	0.16	No break
PAAm (3 M)-MSiO_2_ 216.5 nm	316	19.0	0.15	No break
PAAm (3 M)-MSiO_2_ 311.5 nm	231	11.2	0.11	No break
PAAm (4 M)-MSiO_2_ 117.5 nm	594	42.0	0.20	No break
PAAm (4 M)-MSiO_2_ 216.5 nm	415	21.0	0.22	No break
PAAm (4 M)-MSiO_2_ 311.5 nm	403	20.0	0.17	No break
PAAm (5 M)-MSiO_2_ 117.5 nm	296	17.0	0.29	No break
PAAm (5 M)-MSiO_2_ 216.5 nm	704	38.0	0.44	No break
PAAm (5 M)-MSiO_2_ 311.5 nm	440	21.0	0.25	No break

*Due to the maximum limit of the instruments, the parameter has to be calculated after 24 times of elongation without a break in samples.

### Compressive strength of PAAm-MSiO_2_ hydrogels prepared by varying particle sizes of MSiO_2_ (117.5 nm, 216.5 nm, and 311.5 nm)

**Figure 8:**
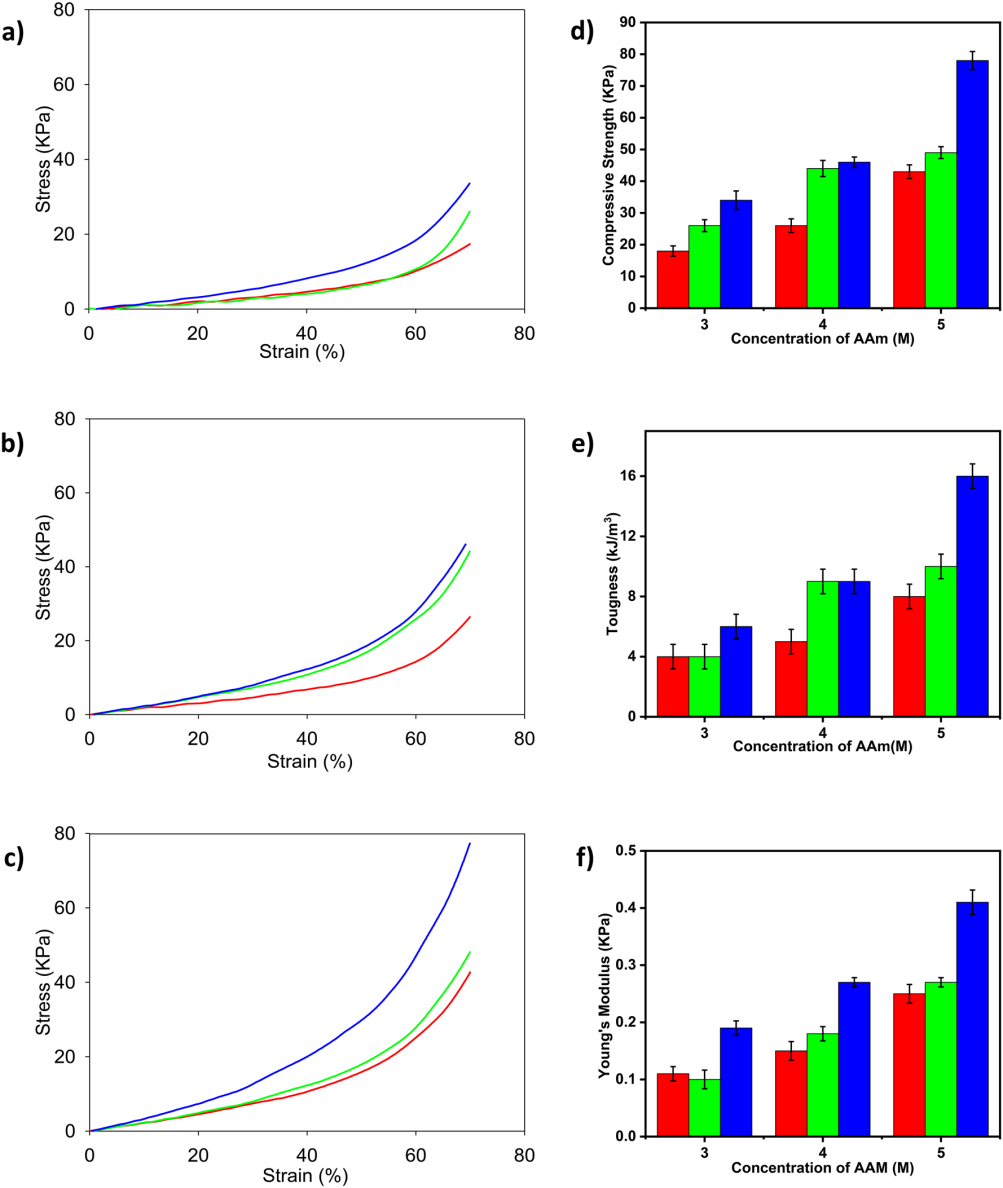
Stress–strain curves produced by compressive strength test of PAAm (3 M)-MSiO_2_ 0.75% (**a**), PAAm (4 M)-MSiO_2_ 0.75% (**b**), and PAAm (5 M)-MSiO_2_ 0.75% (**c**), hydrogels and comparison of compressive strengths (**d**), toughness (**e**), and Young’s modulus (**f**) of PAAm (3 M)-MSiO_2_ 0.75%, PAAm (4 M)-MSiO_2_ 0.75%, and PAAm (5 M)-MSiO_2_ 0.75% hydrogels. Red, green, and blue colors represent MSiO_2_ cross-linker having a diameter of 117.5 nm, 216.5 nm, and 311.5 nm, respectively.

The stress–strain curves for compressive strength of PAAm-MSiO_2_ hydrogels prepared by varying particle sizes of MSiO_2_ (117.5 nm, 216.5 nm, and 311.5 nm) and different concentrations of AAm have been shown in Fig. 8a–c. When stress was applied to the hydrogel, the total energy could be dissipated throughout the homogeneously distributed hydrogel network. The compressive strength, toughness, and Young’s modulus of PAAm-MSiO_2_ hydrogels composed of 0.75% of MSiO_2_ cross-linker were increased when the diameter of MSiO_2_ increased (Fig. 8d–f, Table 6). We have proposed two hypotheses to address the phenomenon. Firstly, the bigger cross-linkers occupy more volume in space resulting increment of the ordered orientation of the polymer networks due to less entanglement probability and thereby provided enhanced mechanical properties. Secondly, the bigger particles are harder and more rigid than the small particles resulting improved compressive strength. When a force was applied, the hydrogel having a larger MSiO_2_ particle size resists more applied force than the gel cross-linked by small particles. As a result, the hydrogels were capable of resisting a tremendous compressive force and showed higher compressive strength, toughness, and Young’s modulus. The interlinkage of the SiO_2_ surface and polymer chain becomes stronger when the particle diameter decreases. Then the strong interaction obliges the polymer chains to enfold tightly to the SiO_2_ particles and makes hydrogel networks tough^37–39^. The high interfacial area between MSiO_2_ particles and AAm polymer matrix guides to a superior bonding between the two phases and provides excellent mechanical strength to the PAAm-MSiO_2_ hydrogels. Experimental investigations have confirmed that the dispersion of particles in the polymer matrix leads to an unprecedented increase in elastic stiffness, starting at a meager volume fraction of particle content^16^.

**Table 6:** Comparison of compressive strength of PAAm-MSiO_2_ hydrogels prepared by varying particle sizes of MSiO_2_ (117.5 nm, 216.5 nm, and 311.5 nm).

Hydrogel code	Toughness (kJ/m^3^)	Compressive strength (kPa)	Young’s modulus (kPa)
PAAm (3 M)-MSiO_2_ 117.5 nm	4	18	0.11
PAAm (3 M)-MSiO_2_ 216.5 nm	4	26	0.10
PAAm (3 M)-MSiO_2_ 311.5 nm	6	34	0.19
PAAm (4 M)-MSiO_2_ 117.5 nm	5	26	0.15
PAAm (4 M)-MSiO_2_ 216.5 nm	9	44	0.18
PAAm (4 M)-MSiO_2_ 311.5 nm	9	46	0.27
PAAm (5 M)-MSiO_2_ 117.5 nm	8	43	0.25
PAAm (5 M)-MSiO_2_ 216.5 nm	10	49	0.27
PAAm (5 M)-MSiO_2_ 311.5 nm	16	78	0.41

## Conclusion

PAAm-MSiO_2_ hydrogel with better mechanical properties than its conventional counterpart PAAm-BIS hydrogels has been reported. The poor mechanical strength of conventional hydrogel is improved by incorporating vinyl-modified SiO_2_ particles as cross-linkers, which get dispersed homogeneously throughout the internal spaces of gel matrix and create strong bonds with AAm polymer network capable of transforming from an intertwined globular shape to coiled shape having the ability to withstand against high compression and stretch. The PAAm-MSiO_2_ hydrogel has very high stretchability even under high applied forces. The mechanical properties of PAAm-MSiO_2_ hydrogel might be regulated accurately by changing the concentration of both monomer and MSiO_2_ cross-linker. The experimental data of our project correlates between the various mechanical parameters of PAAm-MSiO_2_ hydrogels and the particle size of MSiO_2_ cross-linker. The tensile strength, toughness, and Young’s modulus decreased with the increase of MSiO_2_ cross-linker size from 117.5 to 311.5 nm under uniaxial tensile deformation. The PAAm-MSiO_2_ hydrogel prepared by MSiO_2_ having diameter of 117.5 nm exhibited highest tensile strength, toughness, and Young’s modulus during elongation compared with other hydrogels. In contrast, PAAm-MSiO_2_ hydrogel with a large MSiO_2_ particle size demonstrated higher compressive strength, toughness, and Young’s modulus compared with other hydrogels under uniaxial compressive deformation. The report will be a good source for researchers exploring and searching for effective and reliable techniques to improve the mechanical properties of organic and inorganic composite hydrogels and other smart materials.

## Data Availability

The datasets used and/or analyzed during the current study available from the corresponding author on reasonable request.
